# Cost-Effectiveness of Pharmacotherapy to Reduce Obesity

**DOI:** 10.1371/journal.pone.0026051

**Published:** 2011-10-27

**Authors:** J. Lennert Veerman, Jan J. Barendregt, Megan Forster, Theo Vos

**Affiliations:** School of Population Health, The University of Queensland, Brisbane, Australia; University of Ottawa, Canada

## Abstract

**Aims:**

Obesity causes a high disease burden in Australia and across the world. We aimed to analyse the cost-effectiveness of weight reduction with pharmacotherapy in Australia, and to assess its potential to reduce the disease burden due to excess body weight.

**Methods:**

We constructed a multi-state life-table based Markov model in Excel in which body weight influences the incidence of stroke, ischemic heart disease, hypertensive heart disease, diabetes mellitus, osteoarthritis, post-menopausal breast cancer, colon cancer, endometrial cancer and kidney cancer. We use data on effectiveness identified from PubMed searches, on mortality from Australian Bureau of Statistics, on disease costs from the Australian Institute of Health and Welfare, and on drug costs from the Department of Health and Ageing. We evaluate 1-year pharmacological interventions with sibutramine and orlistat targeting obese Australian adults free of obesity-related disease. We use a lifetime horizon for costs and health outcomes and a health sector perspective for costs. Incremental Cost-Effectiveness Ratios (ICERs) below A$50 000 per Disability Adjusted Life Year (DALY) averted are considered good value for money.

**Results:**

The ICERs are A$130 000/DALY (95% uncertainty interval [UI] 93 000–180 000) for sibutramine and A$230 000/DALY (170 000–340 000) for orlistat. The interventions reduce the body weight-related disease burden at the population level by 0.2% and 0.1%, respectively. Modest weight loss during the interventions, rapid post-intervention weight regain and low adherence limit the health benefits.

**Conclusions:**

Treatment with sibutramine or orlistat is not cost-effective from an Australian health sector perspective and has a negligible impact on the total body weight-related disease burden.

## Introduction

Obesity in adults is a global public health concern because excess weight increases the relative risk of disease and mortality [1], [2], [3]. A range of diseases, notably cardiovascular disease, diabetes and a number of cancers, are related to excess weight [4]. High body mass was responsible for 7.5% of the total burden of disease and injury in Australia in 2003 [5].

Several treatment options for obesity exist. Obese persons may lose up to 5 to 8.5 kilograms with diet [6], 1 to 2 kg with exercise [7], 2.5 to 5.5 kg with drugs [8], or 20 to 30 kg with bariatric surgery [9].

Most weight loss drugs are either lipase inhibitors or appetite suppressants. Sibutramine (also known under trade names Meridia or Reductil) is a norepinephrine and serotonin reuptake inhibitor. Sibutramine putatively aids weight loss by suppressing appetite and increasing thermogenesis. Orlistat (also known as Xenical or Alli) is a lipase inhibitor which is assumed to aid weight loss by preventing the digestion and absorption of dietary fat. Following a standard dose of 120 mg three times per day before meals, orlistat inhibits approximately 30% of fat absorption [10].

In Australia, sibutramine has been listed for reimbursement since 2001 for obese patients with an initial body mass index (BMI) greater than or equal to 30 kg per square metre or greater than or equal to 27 kg per square metre in the presence of other obesity-related risk factors (e.g. diabetes, dyslipidemia, hypertension). Up to 2010, sibutramine could be prescribed to patients who have not adequately responded to an appropriate weight-reducing regimen alone (hypo caloric diet and/or exercise) [11], [12]. Orlistat is reimbursed for patients with a BMI greater than or equal to 35 with no known co-morbidities, or a BMI greater than or equal to 30 with co-morbidities (diabetes, ischemic heart disease, psychiatric conditions, and/or hypertension). Patients are eligible for 1 continuous 12-month treatment in a lifetime, and should receive dietetic and weight management advice. Orlistat is discontinued after 3 months if the patient's initial body weight has not been reduced by 2.5 kg or 2.5% (whichever is the lesser), and after 6 months if the loss was less than 5 kg or 5% [13].

Cost-effectiveness is one of the criteria used to decide whether to fund health care interventions. Cost-effectiveness analysis is a form of economic analysis that compares the relative costs and outcomes (effects) of two or more courses of action, one of which is often ‘do nothing’ or ‘business as usual’. In the health field, the outcomes are often expressed in quality-adjusted life years (QALYs) or disability-adjusted life years (DALYs) [14]. Both of these measures combine effects on duration and quality of life, but while the QALY is a measure of health gain, the DALY is a measure of health loss. The DALY concept was developed for the Global Burden of Disease study (GBD) in the early 1990s [15]. To enable comparison of the severity of different diseases, a coherent set of ‘disability weights’ was based on expert opinion. The DALY used in economic evaluations varies from the DALY in burden of disease studies: a) fatal health loss is based on mortality rates in the population of interest rather than being mirrored against a standard life expectancy; and b) the GBD age weights are not used. In fact, the only difference between estimating DALYs and QALYs in our cost-effectiveness studies is the use of the GBD disability weights rather than QALY utility weights.

A recent systematic review of cost-effectiveness analyses of pharmacological anti-obesity treatments found that most published studies reported findings that were within the cost-effective range [16]. However, 11 of the 14 studies were sponsored by the companies that market the drugs, and the 3 independent studies found much higher incremental costs per quality-adjusted life year (an average of €62,000/QALY for independent studies versus €15,000/QALY for industry-sponsored studies). Although attrition rates in anti-obesity pharmacotherapy are high [17], this was not clearly accounted for in any of the studies. The authors of the review concluded that there was a need for independently conducted studies that explicitly address the high discontinuation rates and compare different drugs head-to-head. The present research was carried out independently from industry and evaluates the cost-effectiveness of treatment with sibutramine or orlistat to reduce obesity in Australia, 2003. In addition, it estimates by how much these drugs could potentially reduce the burden of disease due to excess body mass.

The study forms part of the ‘Assessing the Cost-Effectiveness (ACE) Prevention’ project, which was established to provide a comprehensive analysis of the cost-effectiveness of interventions that address the non-communicable disease burden in Australia. The project takes a health sector perspective and applies a lifetime time horizon. The rationale and methodology have been explained elsewhere [18]. Earlier studies reported on weight reducing diets [19], nutrition labelling and a ‘junk food’ tax [20], physical activity interventions [21] and fruit and vegetable interventions [22].

## Methods

### Modelling health outcomes and costs

We constructed a proportional multi-state life table Markov model [23], [24] to calculate health outcomes resulting from a reduction in average body weight in the population. The model simulates and compares two populations in separate life tables: a reference population based on existing levels of morbidity and mortality for 2003 and an intervention population which is identical except that it receives the intervention. Table 1 lists all effectiveness and costing parameters in the model, along with their distributions and sources.

**Table 1:** Input parameters relating to effectiveness and intervention costs.

Parameter	ValueMean (SD/min-max)^a^	Uncertainty distribution	Sources and assumptions	Intervention
Mean reduction in body weight (kg) at 12 months	4.45 (0.426)^b^	Normal	Meta-analysis [10]	Sibutramine
Mean reduction in body weight (kg) at 12 months	2.89 (0.316)^b^	Normal	Meta-analysis [10]	Orlistat
Rate of weight regain in maintenance phase (kg/month)	0.0309 (0.0084)^b^	Normal	Meta-regression [50]; exact mean and SD personal communication with authors.	Sibutramine, Orlistat
Relative risk (RR) of obesity related disease	See Table 2.	Normal (ln RR)	Relative risks by age from CRA project [37].	Sibutramine, Orlistat
Disability weights of obesity related disease	See source.	–	Differs by disease, age and sex; from Australian Burden of Disease 2003 study [5].	Sibutramine, Orlistat
Proportion of target group seen by GP in any 1 year	83,5%	–	[51]	Sibutramine, Orlistat
Proportion of obese patients willing to try weight-reducing drug	50%	–	Authors' estimate; no information available.	Sibutramine, Orlistat
Attrition	48%	–	[52]	Sibutramine
Attrition	33%	–	[52]	Orlistat
Average year-equivalent of drug use per starting participant	0.64	–	[52] Assume 50% of attrition in 1^st^ month, rest at 6 months, on average.	Sibutramine
Average year-equivalent of drug use per starting participant	0.75	–	[52] Assume 50% of attrition in 1^st^ month, rest at 6 months, on average.	Orlistat
Sibutramine120 mg daily (total yearly cost)	$1,467	–	Pharmaceutical Benefit Schedule	Sibutramine
Orlistat 120 mg 3×day (total yearly cost)	$1,486	–	Pharmaceutical Benefit Schedule	Orlistat
Standard GP consultation	$30.20	–	Level B consultation, Item MBS 23, MBS 2003	Sibutramine, Orlistat
GP per hour	$109.36	–	Inner Eastern Melbourne Division of General Practice	Sibutramine, Orlistat
Time spent with GP during initial appt relating to referral or prescription (mins)	10 (9–11)	Triangular	[53]	Sibutramine, Orlistat
Time spent with GP during follow-up visit	25 (20–30)	Triangular	[53]	Sibutramine, Orlistat
Waiting time before individual appointment GP (mins)	30	–	[53]	Sibutramine, Orlistat
Average time to travel TO and FROM meetings (mins)	30 (24–36)	Triangular	Own estimate	Sibutramine, Orlistat
Cost of patient time (per hour)	$17.44	–	Derived from labour force participation [54] and average weekly earnings [55]	Sibutramine, Orlistat
Cost of patient travel (per trip)	$7.45	–	Based on average distance travelled to GP for urban (estimate), regional [56] and remote [57] populations, and Royal Automobile Club Victoria private vehicle reimbursement rate for medium 2–3 L vehicles.	Sibutramine, Orlistat

NB. All costs adjusted to 2003 Australian dollars using Australian health price deflators [35], consumer price index [36] and/or purchasing power parities [58] where relevant.

a For triangular distributions the most likely values are given, with the minimum and maximum values in brackets.

b The value in brackets is the standard error of the mean in the source data, but is used in the model as the standard deviation of the distribution around the change in the population mean of body weight.

#### Interventions

In the model, pharmaceutical intervention with either sibutramine 120 mg or orlistat 3×15 mg daily is offered to all obese Australian adults (21% of men and 23% of women) [25]. Recruitment and prescribing are assumed to be via opportunistic screening by GPs. Since 84% of Australians are seen by a GP in any one year and we (perhaps optimistically) assume 50% of the eligible population would agree to participate, we estimated recruitment at 42% of the target population. We assume a maximum treatment duration of one year and compared against a no-intervention scenario.

#### Weight Change

A recent meta-analysis estimated that sibutramine reduced body mass by 4.45 kg (standard error [SE] 0.426) at the end of 12 months, and orlistat by 2.89 kg (SE 0.316) [10]. A slightly more recent study found very similar results but did not give results specifically at 12 months, which we preferred because it accords well with Australian reimbursement practice and with the structure of our model (which has one-year cycles) [8]. We implicitly assume that adherence in practice was no different from adherence in the trials that were included in the meta-analysis. Consistent with the intent-to-treat analysis used in the primary studies, we apply the weight loss to all persons who started the drug and do not reduce the effect for discontinuation of treatment or non-adherence. Weight which is lost with drugs tends to be regained. For both drugs, we apply the weight regain of 0.385 kg/month observed in the STORM trial with sibutramine [26] to this average weight loss at the end of the intervention year, and assume that no permanent weight loss is achieved.

#### Health effects

Effectiveness is modelled by dividing the populations into 5-year cohorts and simulating each cohort in the life table until all persons have died or reached 100 years of age. Years of life lived are adjusted at each age for time spent in poor health due to disease or injury. The model was implemented in Microsoft Excel 2003 (Microsoft Corp., Redmond, Washington).

The model explicitly simulates nine obesity-related diseases: stroke, ischemic heart disease, hypertensive heart disease, diabetes mellitus, osteoarthritis, post-menopausal breast cancer, colon cancer, endometrial cancer, kidney cancer [4].

For each disease, a simple disease model calculates the effect on prevalence and disease-specific mortality in subsequent years as a consequence of changes in disease incidence [27]. Each disease state is assigned a disability weight (Table 2) based on the Australian Burden of Disease Study [5]. Disease specific changes in prevalence and mortality are linked to the life table and influence total mortality rates and the average health-related quality of life at each age and sex, and therefore the total number of disability-adjusted life years lived by the cohort.

**Table 2:** Disability weights for prevalent diseases, by sex, at baseline [5].

	Male	Female
Colorectal Cancer	0.12	0.11
Breast cancer	-	0.12
Endometrial cancer	-	0.03
Kidney cancer*	0.06	0.06
Ischemic heart disease*	0.04	0.06
Stroke*	0.31	0.31
Hypertensive heart disease	0.09	0.07
Type II Diabetes*	0.08	0.08
Osteoarthritis*	0.05	0.06

*Disability weights used differ by age; weighted average at baseline (2003) is presented.

The reference population is simulated based on the measured body weight distribution in year 1999/2000 [25] and expected future trends in obesity in Australia [28]. The characteristics of the intervention population are the same except that the obese lose a number of kilograms, which lowers the risk of each of the diseases related to excess weight. The change in disease incidence is estimated through Potential Impact Fraction (PIF) calculations [29]. The relative risks used [4], [30] are detailed in Table 3.

**Table 3:** Relative risks of disease per 1 unit increase of BMI [4], [30].

	Age	
Colorectal cancer	<35	1
	35+	1.03 (1.01–1.05)
Breast cancer	<35	1
	35+	1.03 (1.02–1.04)
Endometrial cancer	<35	1.10 (1.07–1.14)
	35+	1.10 (1.07–1.14)
Kidney cancer	<35	1.06 (1.03–1.08)
	35+	1.06 (1.03–1.08)
Osteoarthritis	<35	1.04 (1.03–1.06)
	35+	1.04 (1.03–1.06)
Ischemic heart disease	<35	1
	35–44	1.12 (1.05–1.19)
	45–59	1.10 (1.08–1.14)
	60–69	1.06 (1.03–1.08)
	70–79	1.04 (1.02–1.06)
	80+	1.02 (1.00–1.05)
Hypertensive heart disease	<45	1
	45–59	1.09 (1.03–1.14)
	60–69	1.16 (1.05–1.27)
	70–79	1.12 (1.04–1.21)
	80+	1.06 (1.02–1.11)
Stroke	<35	1
	35–44	1.14 (1.05–1.23)
	45–59	1.10 (1.03–1.16)
	60–69	1.08 (1.03–1.13)
	70–79	1.05 (1.02–1.09)
	80+	1.03 (1.01–1.05)
Type II Diabetes	<35	1
	35–44	1.19 (1.06–1.32)
	45–69	1.14 (1.05–1.23)
	70+	1.10 (1.03–1.16)

NB. Values shown are the mean and 95% confidence intervals.

Estimates of disease incidence and mortality are based on the Australian Burden of Disease 2003 study [5]. In the absence of coherent data on the prevalence of these diseases among the obese, which are needed to define the baseline situation, we generate estimates using the model itself. This requires assuming the population is in a steady state (i.e., ignoring trends) and defining the intervention scenario as all Australians having a BMI over 30 (by using the part of the BMI distribution that exceeds 30 kg.m^2^ and inflating it to the size of the total Australian population). This allows calculating PIF values, which are applied to the Australian incidence rates to estimate disease incidence among obese Australians. Running the model over the lifetime of the youngest cohort then gives the estimates of the baseline prevalence that we need as input for our modelling. The prevalence estimates obtained this way for IHD, stroke and diabetes were comparable to those obtained by analysing measured data from the AusDiab study [25] without the random variation by age (results not shown).

#### Costs

Intervention costs are assessed from a health sector perspective. We include the costs of pharmaceuticals, GP visits, and lifetime health care costs. We separately present results that additionally include participants' travel costs and the cost of time spent visiting the GP. In the base case scenario, participants' time and travel costs are included. Participants' time is valued at 25% of the wage rate, i.e. A$17.44 per hour [31]. Table 1 summarizes the assumptions and costing figures.

Intervention costs include 10 minutes GP time during the initial GP appointment, the cost of medication for up to one year, and an average of 1.3 (sibutramine) or 1.6 (orlistat) medication-related follow-up visits per person starting on medication. This low number of doctor visits is partly due to non-adherence of 48% for sibutramine and 33% for orlistat [17]. Half of the attrition is estimated to occur immediately after prescription (incurring 1 month of drug costs), the rest on average 6 months after starting therapy (incurring 6 months of both drug costs). Based on information of the Pharmaceutical Benefit Schedule, a full year of medication is estimated to cost A$1,467 for sibutramine and A$1,486 for orlistat.

The intervention costs are partially offset by reduced health care expenditure for diseases related to obesity in later years. For cancers, these costs are evaluated for each incident case averted and for the remaining diseases for each prevalent case averted. The literature is divided as to whether health care costs in the years of life that were added by interventions should or should not be included [32], [33], [34]. We present results both ways. Disease treatment costs are drawn from the Australian Institute of Health and Welfare [35] and are detailed in Table 4.

**Table 4:** Average health care costs per prevalent or incident case of disease.

Age	Colon Cancer^a^	Breast Cancer^a^	Endo-metrial Cancer^a^	Kidney Cancer^a^	Ischemic Heart Disease^b^	Stroke^b^	Hypertensive Heart Disease^b^	Type II Diabetes^b^	Osteo-arthritis^b^	All other^c^
Males										
<55	$17,490	–	–	$16,298	$2,962	$2,228	$13,103	$504	$4,431	$1,555
55–64	$17,657	–	–	$16,751	$1,988	$4,942	$24,408	$660	$4,431	$2,828
65–74	$18,164	–	–	$14,748	$1,664	$9,529	$15,048	$763	$4,431	$4,731
75–84	$18,037	–	–	$14,526	$1,512	$12,856	$8,167	$639	$4,431	$7,945
85+	$19,288	–	–	$7,372	$1,394	$16,301	$1,723	$594	$4,431	$13,061
Females										
<55	$17,136	$12,424	$10,665	$15,505	$1,832	$1,161	$22,097	$506	$4,431	$2,009
55–64	$16,349	$10,493	$9,902	$16,363	$1,520	$2,090	$32,044	$759	$4,431	$3,225
65–74	$17,238	$11,609	$14,419	$17,133	$1,595	$5,106	$20,357	$839	$4,431	$4,829
75–84	$17,360	$12,706	$10,497	$17,198	$1,564	$13,137	$9,624	$745	$4,431	$8,197
85+	$16,545	$12,520	$13,402	$12,192	$1,670	$19,679	$1,695	$429	$4,431	$15,078

a Cost per incident case of disease.

b Annual cost per prevalent case of disease.

c Annual cost per person.

NB. Costs are in Australian dollars, adjusted to the year 2003.

All costs are adjusted to real prices in the 2003 reference year using the relevant Health Price Index from the Australian Institute of Health and Welfare [35], or the Consumer Price Index from the Australian Bureau of Statistics [36] if the costs occurred outside the health sector.

#### Calculation of DALYs and ICERs

DALYs averted by the intervention are calculated as the increase in the discounted number of life years lived in the intervention population compared to the reference population, with an adjustment for disability. To show the impact of interventions on the total burden of high body mass in the Australian population, we also express the health gains at the population level as a percentage of the gains that could be achieved under an ideal scenario in which the population has a BMI of 21 (SD 1), which is the theoretical minimum risk scenario used in the World Health Organization's Comparative Risk Assessment study [37], over the rest of their lifetime. We calculate incremental cost-effectiveness ratios (ICERs) as the discounted net cost of the intervention (total cost less cost offsets) divided by the DALYs gained by the intervention, compared to no intervention. Discounting of costs and benefits is at 3% per year [38]. In the ACE Prevention project, interventions with ICERs under A$50,000/DALY (in 2003, A$1 was approximately US$0.67 or €0.58) are considered cost-effective in the Australian context, although in the light of revealed preferences some may consider this a rather low threshold [39].

#### Uncertainty

Uncertainty intervals for DALYs, net costs and the ICER are estimated by Monte Carlo simulation (2000 iterations) using the Excel add-in Ersatz (www.epigear.com). Normal distributions are assumed around the change in average weight that results from each intervention and around the natural log of the relative risk of obesity related disease. A triangular distribution was assumed around a few of the costing components. Importantly, no uncertainty distribution was assumed around the cost of medication, which makes up over 90% of the total intervention costs. This is because both drugs are (or, in the case of sibutramine, were) subsidized to this centrally determined price level. Table 1 lists the parameters with their distributions and values.

#### Sensitivity Analyses

We explore the range of cost-effectiveness outcomes by including patients' time and travel costs, and by including health care costs for unrelated diseases into the cost-offset calculations. To enable comparison with previous studies, we also assess the effect of halving the rate at which weight is regained; of linear weight regain over 3 years following the cessation of therapy; of assuming 23% of the weight loss is permanent; and of including a utility-weight of 0.017 per BMI-unit lost for one year. Also presented are a scenario without discounting, one in which all obese are assumed free of obesity related disease at baseline, and results by age.

## Results

We simulated a total loss of DALYs due to excess body mass over the lifetime of Australians aged 20 years and above in 2003 of 5.8 million (95% confidence range 4.9–6.6 million). Neither of the evaluated interventions averts more than 0.2% of this disease burden (Table 5).

**Table 5:** Cost-effectiveness of sibutramine and orlistat when compared with current practice.

	Sibutramine	Orlistat
**Health impact**		
DALYs averted	11 000 (7 800–15 000)	6 500 (4 500–8 800)
Proportion of total burden averted	0.2%	0.1%
**Costs**		
Intervention Cost ($million)		
Health care sector	1 500 (1 500–1 500)	1 500 (1 500–1 500)
Patient time and travel	44 (41–48)	56 (51–60)
Healthcare costs ($million)		
Cost offsets	−99 (−130–−73)	−59 (−77–−41)
Healthcare costs in added years of life	57 (42–74)	34 (24–44)
**Cost-effectiveness**		
$/DALY		
Intervention costs+cost offsets	130 000 (93 000–180 000)	230 000 (170 000–340 000)
+ patient time and travel costs	130 000 (96 000–190 000)	240 000 (170 000–350 000)
+ costs of unrelated health care in added years of life	140 000 (100 000–190 000)	240 000 (180,000–350 000)
Probability cost-effective at $50,000/DALY		
Intervention costs+cost offsets	0%	0%
+ patient time and travel costs	0%	0%
+ costs of unrelated health care in added years of life	0%	0%

NB. Values for health impacts and costs are means and 95% uncertainty intervals, rounded to two significant figures. Cost-effectiveness ratios are ‘ratios of means’ [59] with 95% uncertainty ranges and are expressed in Australian dollars per disability-adjusted life year, referenced to the year 2003.

With ICERs between A$93 000 and A$350 000 per DALY, treatment of obesity with sibutramine or orlistat is not cost-effective in any of the costing scenarios (Table 5).

The sensitivity analysis (Table 6) shows that halving the rate at which weight is regained does not materially change the results. Assuming weight is regained linearly over 3 years doubles the health impact but does not result in ICERs in the cost-effective range. In contrast, the assumption that 23% of the weight lost is not regained dramatically improves the cost-effectiveness ratios. In this scenario, treatment with either drug would likely be considered cost-effective. Attributing a utility value to BMI-loss per se also greatly improves the cost-effectiveness ratios. This adds so many QALYs to the DALYs we used in our standard methods that it pushes sibutramine into the cost-effective range, with orlistat coming close. Discounting has significant impact on the cost-effectiveness ratios but discounting at 0% does not change the conclusions. Neither does age. In our model, the benefits in the 20–29 age category are zero because the risk of disease below age 35 was assumed unrelated to BMI (Table 3). Above that age the cost-effectiveness improves up to age 60–69, but only to $88 000 per DALY for sibutramine and $160 000 for orlistat. Above that age it deteriorates again due to lower relative risks and the lower life expectancy of the additional survivors. The starting prevalence of obesity-related diseases had no material influence on the results, probably because the model does not vary the risk of incident disease based on medical history.

**Table 6:** Results of univariate sensitivity analysis.

	Sibutramine		Orlistat	
	DALYs averted	$/DALY	DALYs averted	$/DALY
Base case	11 000	140 000	6 500	240 000
Weight regain halved	15 000	100 000	8 200	190 000
Linear wt regain over 3 yrs	19 000	75 000	13 000	120 000
23% wt loss permanent	71 000	18 000	49 000	29 000
Incl. utility for BMI-loss*	56 000	27 000	32 000	63 000
No discounting	18 000	84 000	11 000	150 000
Disease-free at baseline	13 000	120 000	7 500	210 000
Age 20–29	0	∞	0	∞
Age 30–39	910	290 000	530	530 000
Age 40–49	2 900	110 000	1 700	200 000
Age 50–59	3 400	100 000	2 000	180 000
Age 60–69	2 400	88 000	1 400	160 000
Age 70–79	1 300	110 000	740	200 000

NB. Values for DALYs averted are means, while those for A$/DALY are ratios of means, rounded to two significant figures. Cost-effectiveness ratios are in Australian dollars per disability-adjusted life year, referenced to the year 2003. The ICERs include the costs of participants' time and travel and the health care costs in added years of life.

*In the column titled ‘Incl. utility for BMI-loss’, the calculations have been made after adding the BMI-related QALYs that were gained to the DALYs averted.

## Discussion

By the criteria used in this study, treating obese persons with sibutramine or orlistat is not cost-effective in the Australian setting and has a negligible impact on the burden of disease due to excess body mass.

### Comparison of results with other studies

In contrast to our findings, the majority of previous studies have concluded that sibutramine and orlistat are cost-effective. Our cost-effectiveness ratios for sibutramine and orlistat are over twice as high as those in earlier studies [16]. Two factors explain part of this difference.

Firstly, in many previous studies the rate at which lost weight is regained is more optimistic than in ours. Many studies assume an arbitrary linear regain over 3 or 5 years, while this study uses an empirical rate of regain [26] which results in regain of all lost weight within 2 years, on average. One study assumed that 23% of the weight loss lasts a lifetime [40], based on an older review of dietary interventions (rather than pharmaceutical interventions) [41]. Our sensitivity analysis shows that the rate of weight regain has a modest impact on the results, but the results are very sensitive to assumptions around the permanency of weight loss.

Secondly, many studies attribute a (substantial) ‘utility’ gain to weight loss per se, while this study only includes reductions in disease-related quality and length of life. We thus ignore any improvements in mental health, fitness and wellbeing that may be the result of weight loss, even in the absence of changes in disease status. Adding such effects would improve the cost-effectiveness estimates. A number of previous studies [42], [43], [44], [45], [46] use a utility value of 0.017 per BMI unit lost per year from a pharma-sponsored study that used data from an RCT [47]. This value is high; it would imply that a person with a BMI of 38 and a quality of life of 80% of perfect health could achieve a quality of life better than perfect when reaching normal weight. Studies that use data from trials of weight reducing interventions to derive quality of life values for body mass changes may measure not only health-related quality of life, but also the satisfaction of achieving weight loss or the disappointment of having failed to lose weight, which in our study is not taken into account. In the study from which the 0.017 utility value was derived, the measurement of persons health status with a visual analogue scale was preceded by questions that framed ‘health’ in terms of physical attractiveness, social functioning, health distress and emotions [47]. This may have influenced participants' responses.

### Limitations

Consistent with our health sector perspective, our study does not take into account any increases in productivity that may result from reduced BMI. Including these benefits would improve the cost-effectiveness of both interventions for the working-age population. However, this would be partly or completely offset by older persons, who are generally less productive, living longer.

Persons taking weight reducing drugs are also advised to follow a diet. Our analysis did not include a diet, but since the diet recommendation would apply equally to the intervention and the reference group, this makes no difference to the results.

We did not model any persons discontinuing therapy because of insufficient weight loss, but it seems very unlikely that such stopping rules would improve the cost-effectiveness enough to make treatment with either sibutramine or orlistat cost-effective. A quick back-of-the-envelope calculation shows that even if we cost only half of the persons after 3 months but retain the health effect of the full cohort, the ICERs are around A$85 000 for sibutramine and A$160 000 for orlistat.

On the effect side, we assumed the attrition for drug intervention would be the same as in trials, which is probably too optimistic. No additional costs, health risks or loss of quality of life were included for side-effects of the drugs (mainly increased blood pressure and heart rate for sibutramine and gastrointestinal complaints for orlistat). Quite aside from considerations of efficiency, sibutramine was recently shown to increase the risk of cardiovascular disease among subjects with pre-existing cardiovascular conditions [48]. On that basis, its licence has been withdrawn in Australia [12] and in the European Union [49].

### Conclusion

Given the expected rates of weight regain and the high costs of medication, the implementation of the pharmaceutical interventions for primary prevention of obesity-related disease in Australia is unlikely to offer value for money. Based on current evidence, the overall effect of these pharmaceuticals on the obesity-related burden of disease is negligible.
